# Potential Energy Distribution of Redundant Cable-Driven Robot Applied to Compliant Grippers: Method and Computational Analysis

**DOI:** 10.3390/s19153403

**Published:** 2019-08-02

**Authors:** Alejandro Rodriguez-Barroso, Roque Saltaren, Gerardo A. Portilla, Juan S. Cely, Oz Yakrangi

**Affiliations:** Centro de Automática y Robótica, Universidad Politécnica de Madrid, C/José Gutiérrez Abascal 2, 28006 Madrid, Spain

**Keywords:** parallel robots, kinematics, redundancy, cable-driven robotics, grasping

## Abstract

Cable-driven parallel robots with a redundant configuration have infinite solutions for their cable tension distribution to provide a specific wrench to the end-effector. Redundancy is commonly used to increase the workspace and stiffness or to achieve secondary objectives like energetic minimization or additional movements. This article presents a method based on energy distribution to handle the redundancy of cable-driven parallel robots. This method allows the deformation and tension of each link to be related to the total energy available in the parallel robot. The study of energy distribution expression allows deformation, tension, and position to be combined. It also defines the range of tension and deformation that cables can achieve without altering the wrench exerted on the end-effector. This range is used with a passive reconfigurable end-effector to control the position of two grippers attached to some cables which act as compliant actuators. The relationship between the actuators’ energy and their corresponding gripper positions is also provided. In this way, energy measurement from the actuators allows the grasping state to be sensed. The results are validated using multibody dynamic software.

## 1. Introduction

Cable-driven parallel robots (CDPR) [1,2,3] are a kind of parallel robot whose links that connect the end-effector with actuators are cables. Because they bend under compression forces, they need to be under tension to impose their kinematic restriction. The structure and modeling of CDPR is similar to parallel robots with rigid links and grasping [4] due to the unidirectional restriction provided by cables. 

**Figure 1:**
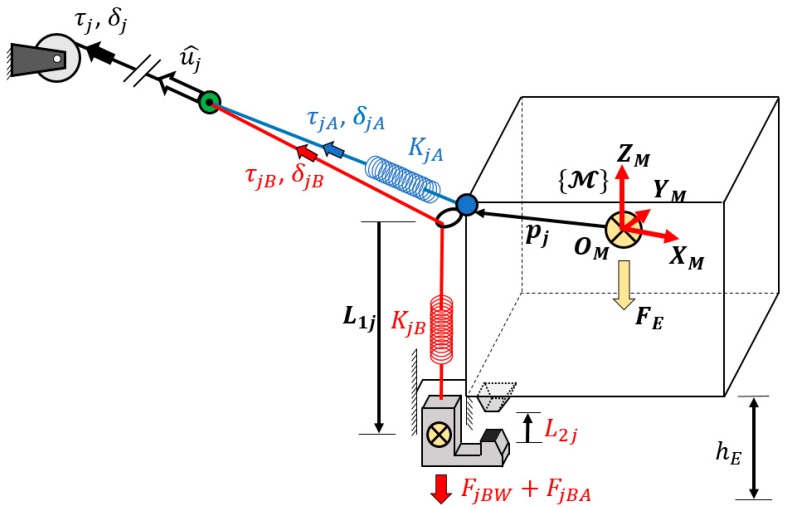
Scheme of the proposed end-effector with a grasping tool based on a reconfigurable end-effector. One cable provides the energy for the tension distribution of the main body and for the displacement of the grasping tool. The cable is coiled around a winch that is able to deploy the desired amount and to impose the desired tension.

One of the main differences between a CDPR and a usual parallel robot [5] is the flexibility and elasticity of the cables [6]. This characteristic is used to control reconfigurable end-effectors with compliant actuators, as seen in [7], where the actuation of a single cable exerts an influence on two different bodies. The scope of this article is to use energy analysis to simplify the resolution of this problem and apply it to the movement of two grippers attached to two cables. Each one has the configuration shown in Figure 1.

In [8,9,10], a cable model is developed. In [11], Behzadipour et al. describe the conditions of stability considering stiffness and internal forces in the cables of CDPR. The control of elastic parallel robots is studied in [12]. In [13], a compensator of cable elasticity is proposed, while the main uncertainties due to the elasto-geometric model are listed in [14]. An elastic model is related to the appearance of vibrations, as seen in [15], which also requires a method for handling vibrations, as seen in the adaptive method of [16] or the active vibration in modal space proposed in [17], the robust PID, as in [18], or mechanical elements, like reaction wheels [19]. This vibration consideration can be seen in relevant prototypes like FAST [20] or Cogiro [21].

CDPRs can be classified according to their number of cables (m). If the end-effector has n degrees of freedom and it has n or less cables, they are underconstrained, because it is not possible to constrain the n degrees of freedom of the end-effector. If there are n + 1 or more cables, the robot is redundant and there exists the possibility to fully constrain it by using the cables [22,23].

Redundant parallel robots were analyzed by Gosselin et al. [24,25]; in the workspace, the effect of six cables at a time was studied in [26]; and the possibility of handling secondary tasks with the redundant actuators appears in [27,28]. In [29,30], an agile algorithm is presented to set the geometric region that defines all feasible tension distributions. All tension distributions defined provide the same force distribution in the end-effector, Lamaury, Gouttefarde et al. provide a useful real-time capable tool to handle CDPR with a degree of redundancy of 2. One commonly used tension configuration comes from the minimization of cable tension by using the Moore–Penrose Pseudoinverse [31]. This kind of optimization has the risk of providing negative tension values that are not feasible for CDPR, so in [32], Borgstrom et al. describe the use of a condition that provides a safe tension distribution, far from faulty regions. 

### 1.1. Contribution

The contribution of this article is the proposal of a CDPR that is able to use its redundancy to specify the aperture of some grippers. This reconfigurable end-effector aims to be passive, having all its electronics, power sources, and actuators situated far from the moving platform, making it suitable for working in harsh environments like underwater, nuclear plants, fires, or outer space. Due to this configuration, the movement of the gripper needs to be sensed by using those measures taken from the drivers and sensors situated outside the robot workspace. This device could be conceived as one application for the secondary tasks that can be performed by the redundant kinematic structure of this elastic parallel robot. More than one gripper can be attached considering the degree of redundancy.

The aperture of the grippers is related to measures like the cable tension, spring deformation, platform position, or friction, so in this article, a method based on the energy spent by the actuators when grasping is proposed to consider all these measures in one value. The energy analysis also allows the amount of energy that the gripper is exerting to the handled object to be known, which is useful for stable and optimum grasping, as shown in [33,34]. Also, it is possible to know the available range of movement of the gripper due to the restrictions of the CDPR. This method is based on the potential energy stored in the robot considering the elasticity of the cables and the springs attached to them. A close-form expression of the energy stored in the CDPR is defined. This expression relates the energy of any cable or all of the robot with the redundant variables defined in [29,30]. 

By considering the energy of the robot and the energy of the cables, it is possible to directly impose the desired energy of any cable and to know the range of energy that each cable can handle for a given end-effector set of forces. This energy analysis allows the range of movement of those cables that exert their actuation on more than one body of the end-effector to be known. In Figure 1, a compliant actuator is proposed based on the previous developments shown in [7]. This mechanism takes advantage of the system redundancy to move a gripper attached to the main body. The proposed method is applied to this system in order to provide the movement of the end-effector and also the movement of a grasping tool attached to it by using energy distribution. The main advantage of energy consideration in comparison with usual tension distributions is the knowledge of the available range of movement of this gripper and other grippers that could be attached to other actuation cables. In addition, by knowing the expression for energy, an analysis of its shape is done. With this knowledge, it is possible to set the direction of the energy boundaries imposed in cables.

The energy method allows this magnitude to be used to consider either the tension or the linear elongation of the cable or even other physical effects such as electromagnetics. Some methods based on energy have recently been used to solve the forward kinematics, as seen in [35,36]. 

The imposed values of tension in redundant cables could be used to set softer variations in the torque of the motor or for modifying the stiffness of the links if they have nonlinear springs. If the linear deformation is imposed, it can be used for controlling reconfigurable end-effectors, like in [7]. 

### 1.2. Mechanism Description

The end-effector can move inside its workspace by pulling from all the cables attached to it from the inertial frame. Its position is defined by the geometric relations, assuming that the cable model is a linear spring defined by the actual spring that is between the cable and the end-effector (blue cable in Figure 1). The end-effector speed is obtained from the inverse kinematics. The actuators, situated outside the workspace, coil each cable by defining their deployed length and applying the desired tension. Parallel robots’ kinematics developments can be seen in [5], while the basic specific relationship with cable-driven robots can be found in [4].

**Figure 2:**
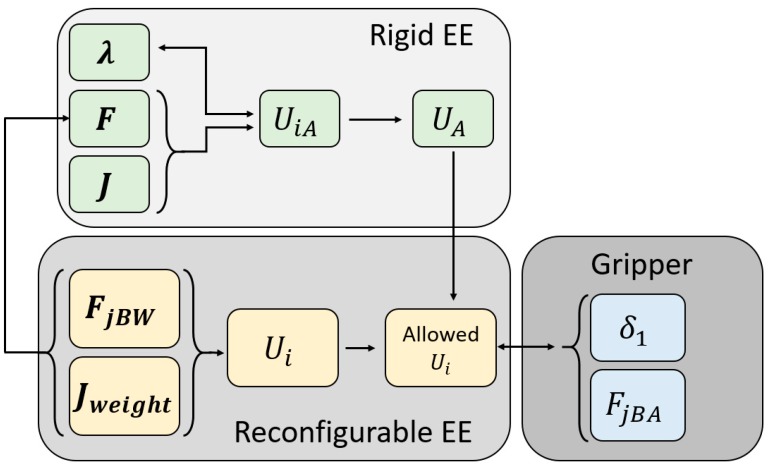
Scheme of the proposed methodology for obtaining the aperture and force exerted by the gripper of the reconfigurable end-effector by considering the boundaries imposed by the rigid end-effector model.

Some of these cables, as much as the redundancy degree, have another cable attached to them (red cable of Figure 1). Due to the ability to control the deformation of the main cable without altering the end-effector, it is possible to move the secondary cable. In this way, one part of a gripper can be situated at the end of the secondary cable, and with the secondary cable movement, the gripper can be opened or closed attending to the distance $L_{2j}$ of Figure 2.

The gripper weight, $F_{jBW}$, will define a new set of requirements for energy distribution. The same method is applied when the gripper grasps an object of known mass. This additional weight can be added to the term $F_{jBA}$, which includes the cable weights and frictions. For those objects of unknown weight, an estimated weight can be added with the following precaution: if the stimulated weight is lower than the real weight, the gripper cannot close, and if the estimation is higher, the closure force will be higher than the calculated force. This decision should be made considering the object toughness. 

Finally, as mentioned in [37], this kind of mechanism has seven different kinds of singularity. Due to the variation in the degrees of freedom and the Jacobian matrix rank, it is recommended to use those secondary tasks in regions far from those singularities.

### 1.3. Article Scheme

The first section defines the theoretic background and the proposed mechanism that take advantage of the CDPR redundancy. The objectives and contribution of the article and the scheme of the proposed methodology for sensing the gripper behavior are set. The second section begins with an analysis of the CDPR with a rigid end-effector from the energy point of view, providing a close form expression to handle the energy with the redundancy, as shown in the first block of Figure 2. After this, the expressions for the reconfigurable end-effector and the influence of the gripper in the behavior of the rigid-end effector are defined, as seen in the second block of Figure 2. The end of this section shows the relation between the actuator energy and the behavior of the gripper, providing a way of sensing the gripper state by using the measures given by the motor and being able to impose the desired position or force values. Section 3 presents the theoretical results of the energy distribution and its relation with the gripper performance. Section 4 compares the previous results with simulations performed using the multibody dynamic software MSC ADAMS. 

## 2. Methodology

The expression for the tension distribution in a redundant CDPR is defined in [30] as

(1) $$\tau ={\left(J^{T}\right)}^{†}F_{ext}+N\left(J\right)\lambda$$

where ${\left(J^{T}\right)}^{†}$ is the (*m* × *n*) matrix corresponding to the pseudoinverse Moore–Penrose of the transpose of the Jacobian matrix. $F_{ext}$ is n-vector of the external wrench applied to the end-effector. N(J) is the (*m* × *r*) matrix of the nullspace of the Jacobian matrix, and λ is an r-vector of values over the basis defined by the nullspace.

### 2.1. Potential Energy of Cables

The potential energy of one *i*-cable is defined considering the model of a cable as a linear spring with stiffness *k_i_*. The energy stored in each spring is defined as

(2) $$U_{i}=\frac{k_{i}\delta_{i}^{2}}{2}$$

where *δ_i_* is the linear deformation of the *i*-cable. This linear deformation is proportional to the mechanical tension of the *i*-cable, as seen in the following equation:

(3) $$\tau_{i}=k_{i}\delta_{i}.$$

It is possible to obtain the potential energy stored in the *i*-cable related to its mechanical tension by combining Equations (2) and (3):

(4) $$U_{i}=\frac{\tau_{i}^{2}}{2k_{i}}.$$

In Equation (1), the nullspace is considered in order to modify the tension distribution along all cables by maintaining the constant value of the resultant wrench in the end effector. This freedom provided by the redundant configuration of the robot can be reflected in the value of the potential energy stored in each one of the cables. In order to clarify the equations, Equation (1) is rewritten in a simpler notation:

(5) τ=B+Aλ

where

(6) $$A=\left(\begin{array}{cccc} a_{11} & a_{12} & \cdots & a_{1r}\\ a_{21} & a_{22} & \cdots & a_{2r}\\ \vdots & \vdots & \ddots & \vdots \\ a_{m1} & a_{m2} & \cdots & a_{mr} \end{array}\right)$$

(7) $$B=\left(\begin{array}{c} b_{1}\\ b_{2}\\ \vdots \\ b_{m} \end{array}\right).$$

By combining Equations (4) and (5), the expression of the potential energy for the *i*-cable in function of the nullspace variables is obtained:

(8) $$U_{i}=\frac{{\left(b_{i}+\sum_{j=1}^{r}a_{ij}\lambda_{j}\right)}^{2}}{2k_{i}}\;$$

By solving the squared term and simplifying the previous equation, the following expression is obtained:

(9) $$U_{i}=\frac{\lambda^{T}P_{i}\lambda +Q_{i}^{T}\lambda +R_{i}}{2k_{i}}$$

with

(10) $$P_{i}=\left(\begin{array}{cccc} a_{i1}^{2} & a_{i1}a_{i2} & \cdots & a_{i1}a_{ir}\\ a_{i1}a_{i2} & a_{i2}^{2} & \cdots & a_{i2}a_{ir}\\ \vdots & \vdots & \ddots & \vdots \\ a_{ir}a_{i1} & a_{ir}a_{i2} & \cdots & a_{ir}^{2} \end{array}\right)$$

(11) $$Q_{i}=2\left(\begin{array}{c} b_{i}a_{i1}\\ b_{i}a_{i2}\\ \vdots \\ b_{i}a_{ir} \end{array}\right)$$

(12) $$R_{i}=b_{i}^{2}$$

With Equation (9), it is possible to vary the energy stored in each cable without exerting any influence on the total wrench of the end effector. In order to describe the potential energy stored in all the cables, the m equations corresponding to the potential energy of each cable seen in (8) need to be merged into:

(13) $$U=\;\sum_{i=1}^{m}U_{i}.$$

By substituting Equation (9) into Equation (13), the expression for the total energy stored in all cables in the function of the nullspace variables is

(14) $$U=\frac{1}{2}\left(\lambda^{T}\left(\sum_{i=1}^{m}\frac{P_{i}}{k_{i}}\right)\lambda +\left(\sum_{i=1}^{m}\frac{Q_{i}}{k_{i}}\right)\lambda +\sum_{i=1}^{m}\frac{R_{i}}{k_{i}}\right).$$

The potential energy stored in the cables is a function of the dimension and position of the robot, the wrench applied to the end effector, the cable stiffness, and the tension distribution along the cables:

(15) $$U=U\left(J,\;F_{ext},\;\lambda ,\;k\right).$$

### 2.2. Energy Boundaries

Each cable is limited to a certain range of tensions higher than zero. This range of tensions is denoted as

(16) $$0<\tau_{min}<\tau <\tau_{max}.$$

The minimum and maximum tension are chosen for working in the linear part of the elastic equation of the spring. Tensions lower than $\tau_{min}$ do not produce elongation, while tensions higher than $\tau_{max}$ make the spring get into the plasticity zone. Those tension value limits can be converted into energy by using the stiffness of each cable, as seen in Equation (4):

(17) $$U_{i}{}_{min}=\frac{\tau_{i_{min}}^{2}}{2k_{i}}$$

(18) $$U_{i}{}_{max}=\frac{\tau_{i_{max}}^{2}}{2k_{i}}$$

It is possible to obtain the tension distribution that makes the cable achieve its maximum or minimum tension. By substituting Equations (17) and (18) into Equation (9), the expressions of $\Lambda_{i_{\max}},\Lambda_{i_{\min}}\subset {\mathbb{R}}^{r}$ are obtained, which represent the nullspace boundaries:

(19) $$U_{i}{}_{min}=U_{i}\to \Lambda_{i_{min}{}_{j,\ldots r}}\left(\lambda \right)=0$$

(20) $$U_{i}{}_{max}=U_{i}\to \Lambda_{i_{max}{}_{j,\ldots r}}\left(\lambda \right)=0$$

The result of this substitution is 2r hypersurfaces, r for $\Lambda_{i_{\max}}^{*}$, and r for $\Lambda_{i_{\min}}$. These hypersurfaces correspond to those regions of the nullspace that generate a tension distribution that exerts its maximum and minimum potential energy in the *i*-cable, respectively. In order to consider only those values that the *i*-cable can handle by pulling, it is necessary to take one value of each hypersurface and check if all the tension values are positive by using Equations (1) or (5). Each one of the 2r border hypersurfaces, $\Lambda_{i}{}_{min},\;\Lambda_{i}{}_{max}\subset {\mathbb{R}}^{r}$, corresponding to each *i*-cable, set the boundaries for the feasible potential energy hypersurface, $\Omega_{i}$, which is defined in the ${\mathbb{R}}^{r+1}$ space. The added dimension represents the value of the *i*-cable potential energy over the r nullspace variables. In order to have a clearer view of this energy shape, the energy is projected over any two nullspace variables ($\lambda_{\alpha },$ $\lambda_{\beta }$). That ${\mathbb{R}}^{3}$ surface corresponds to a parabolic cylinder, as demonstrated in Appendix A. This means that it is possible to represent the feasible potential energy of each cable by projecting it into each pair of nullspace variables. The hyper-surfaces $\Lambda_{i}{}_{min}$ and $\Lambda_{i}{}_{max}$ divide the ${\mathbb{R}}^{r+1}$ space into two different regions: the feasible region and the regions where the tension cable is outside the desired range.

If the potential energy of the *i*-cable is considered to depend only on the value of two nullspace variables ($\lambda_{\alpha },$ $\lambda_{\beta }$), the parabolic cylinder has quadratic terms that can be linearized by rotating the quadric and aligning it with the axis of the variables ($\lambda_{\alpha },$ $\lambda_{\beta }$). This is made by calculating the eigenvectors of the matrix of quadratic terms $P_{i}$. The diagonalized matrix is

(21) $$P_{i}^{*}=\left(\begin{array}{ccc} a_{i\alpha }^{2}+a_{i\beta }^{2} & 0 & 0\\ 0 & 0 & 0\\ 0 & 0 & 0 \end{array}\right)$$

with only one non-null eigenvalue. The corresponding eigenvectors, expressed as the changing base matrix, are

(22) $$V^{*}=\left(\begin{array}{ccc} -\frac{a_{i\alpha }}{a_{i\beta }} & \frac{a_{i\beta }}{a_{i\alpha }} & 0\\ 1 & 1 & 0\\ 0 & 0 & 1 \end{array}\right).$$

It is seen that in Equation (23), there is only one non-null eigenvalue whose eigenvector is $V_{1}^{*}=\left[-\frac{a_{i\alpha }}{a_{i\beta }},\;1,\;0\right]$. This eigenvector defines the direction of the lines that contain the improper centers of the quadric. This direction coincides with those lines that contain the points with the same value of potential energy. In this way, the projected direction of the lines over $\lambda_{\alpha }$ and $\lambda_{\beta }$ of the maximum and minimum potential energy are defined by the eigenvector corresponding to the non-null eigenvalue $V_{1}^{*}$. Indeed, any allowed value of potential energy in any cable produces a line of valid points in the nullspace with the direction $V_{1}^{*}$ when the energy is defined over any two variables ($\lambda_{\alpha },$ $\lambda_{\beta }$).

Finally, the total energy stored in all the cables defines a hypersurface, Ω, that corresponds to an elliptic paraboloid, as seen in Appendix B, when it is projected into any pair of nullspace variables. The feasible region for the total potential energy is defined by the intersection of the m hypersurfaces that correspond to each i-cable:

(23) $$\Omega =\cap_{i=1}^{m}\Omega_{i}.$$

### 2.3. Selection of Single Cable Energy

It is possible to set any desired value to the r nullspace variables **λ** in order to set the corresponding desired potential energy values in each cable. For each variable **λ** whose value is established, the volume of the total potential energy feasible region, Ω, is reduced because, by setting the value of a nullspace variable, the dimension corresponding to that variable collapses into a point. 

The selection of the energy of each cable can be used for multiple purposes, for example, setting a desired tension in a cable or setting a desired linear deformation in its springs. Each imposed cable energy value restricts one of the available nullspace variables, so only r imposed values of tension or deformation can be set as the desired value. 

The region of the nullspace of the *i*-cable that provides its desired potential energy is obtained by using Equation (9) in a similar way as Equations (19) and (20):

(24) $$U_{i}{}_{desired}=U_{i}\to \Lambda_{i_{desired_{1,\ldots ,\left(r-1\right)}}}^{*}\left(\lambda \right)=0.$$

Considering the shape of the quadric already analyzed for ${\mathbb{R}}^{3}$ for the potential energy of a cable, the line of all nullspace values that provides a certain potential energy in a cable can be obtained directly. Once the desired value of potential energy is set, one point of this line is provided by Equation (24), while the direction of the line is provided by Equation (22).

Once the restrictions have been applied, the polytope that establishes the cable tension distribution loses one dimension, shrinking the feasible workspace in the nullspace, and the remaining degrees of freedom can be considered for setting new values of potential energy in other cables.

### 2.4. Energy Analysis of the Reconfigurable End-Effector

The method presented in previous sections allows energy or deformation to be imposed in any cable and to determine the available range of elastic energy that any other cable can store without altering the end-effector wrench. 

In this section, the mechanism proposed in Figure 1 is analyzed to control the movement of the gripper. This mechanism is based on the CDPR with compliant actuators shown in [7] and allows two different bodies of the same end-effector to move by taking advantage of the redundancy and elasticity of the cables. The application of the proposed method determines the range of movement of one or several grippers to identify whether it is possible to close it and to determine the size of object that the gripper can handle for certain positions and the wrench applied to the end-effector. This gripper has its movement restricted to 1 DoF. A vertical linear guide attached to the main body of the end-effector imposes this restriction.

In Figure 1, the energy applied by the motor to cable *j* is exerted on cables $j_{A}$ and $j_{B}$. While $j_{A}$ imposes its tension to the end-effector, $j_{B}$ moves the gripper. The initial displacement $L_{2}$ is measured when the tension in $j_{A}$ is $\tau_{min},$ and the relation between cables deformation and gripper movement is defined by

(25) $$\delta_{j}=\delta_{jA}=\delta_{jB}$$

while the relation between each cable tension is

(26) $$\tau_{j}=\tau_{JA}+\tau_{JB}=\tau_{JA}+∥F_{jBW}^{∥}∥+F_{jBA}$$

where $∥F_{jBW}^{∥}∥$ is the weight of the gripper projected over the hanging cable. Cable $j_{B}$ turns around a ring or a pulley whose constant friction term can be added to $F_{iBW}$ with $F_{jBA}$, that is, the term related to the pulley friction and cable weight.

(27) $$F_{jBW}^{∥}=-F_{jBW}\left(\hat{k}\cdot {\hat{k}}_{M}\right){\hat{k}}_{M}=F_{jBW}\Omega$$

(28) $$F_{jBW}^{⊥}=-F_{jBW}\hat{k}-F_{jBW}^{∥}.$$

**Figure 3:**
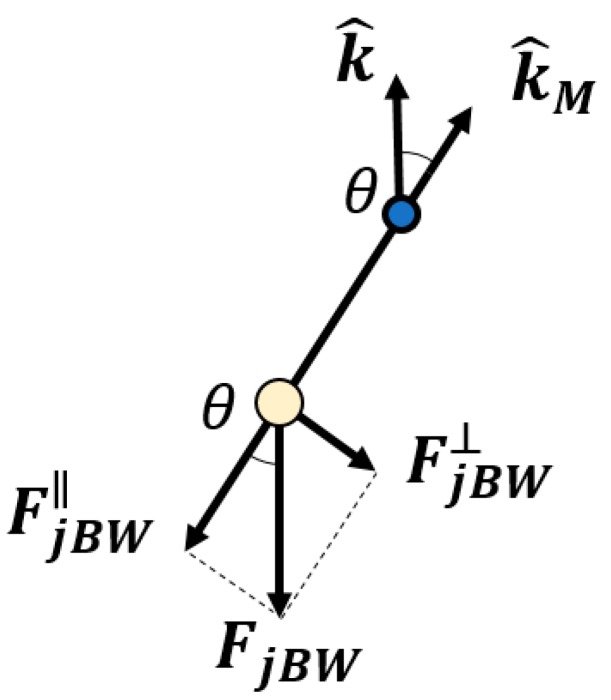
Weight force acting on the gripper. It is projected in parallel to the cable, imposing the tension $\tau_{JB},$ and perpendicular to the cable, acting on the main body of the end effector.

The compliant actuator exerts the tension from $j_{A}$ on the main body of the end-effector as well as the force due to the gripper weight, as represented in Figure 3. The force exerted by this compliant actuator to the main body at point $p_{j}$ (blue dot of Figure 1) is

(29) $$F_{jE_{1}}=\tau_{jA}\hat{u_{i}}+\tau_{jB}\hat{u_{i}}+F_{jBW}^{∥}+F_{jBA}.$$

In addition, the force exerted at point ($p_{j}+L_{1})$ by the weight of the gripper is

(30) $$F_{jE_{2}}=F_{jBW}^{⊥}.$$

It is assumed that $j_{A}$ and $j_{B}$ are parallel. Mechanically, this assumption can be built by using a ring with a small radius or even by passing cable $j_{B}$ inside the coils of the spring $K_{A}$. Due to the low displacement and low weight of the gripper with respect to the main body, $∥L_{1}∥$ is assumed to be constant for all values of $L_{2}$:

(31) $$\left(L_{2}+\max(\delta_{jB})\right)-\left(L_{2}-\min\left(\delta_{jB}\right)\right)\ll ∥L_{1}∥$$

The structure matrix ($J^{T})$ used in Equation (1) can be written considering this compliant actuator as

(32) $$F_{E}=J^{T}\left(\begin{array}{c} \tau_{i}\\ \vdots \\ \begin{array}{c} \tau_{m} \end{array} \end{array}\right)+J_{weight}^{T}\left(\begin{array}{c} \begin{array}{c} \begin{array}{c} F_{jBW}\\ \vdots \\ F_{rBW} \end{array} \end{array} \end{array}\right).$$

The gripper weight is included so the *m* actuated cables define the well-known structure matrix ($J^{T}),$ while the influence of the gripper weight in the main body of the end-effector is defined by ($J_{weight}^{T})$. For a totally constraint mechanism, the maximum number of grippers is *r*. The structure matrix is

(33) $$J^{T}=\left(\begin{array}{ccc} \hat{u_{i}} & \ldots & \begin{array}{c} \hat{u_{m}} \end{array}\\ p_{i}\times \hat{u_{i}} & \ldots & \begin{array}{c} p_{m}\times \hat{u_{m}} \end{array} \end{array}\right)$$

where Equation (26) has been used to set Equation (32) as a function of $\tau_{j}$ and $F_{jBW}$. The wrench applied by the gripper weight to the end effector is related to

(34) $$J_{weight}^{T}=\left(\begin{array}{c} \Omega +\left(-\hat{k}-\Omega \right)\\ \begin{array}{c} p_{j}\times \end{array}\Omega +\left(p_{j}+L_{1j}\right)\times \left(-\hat{k}-\Omega \right) \end{array}\right)$$

By simplifying Equation (34), we obtain

(35) $$J_{weight}^{T}=\left(\begin{array}{c} -\hat{k}\\ \begin{array}{c} p_{j}\times (- \end{array}\hat{k})+L_{1j}\times \left(-\hat{k}-\Omega \right) \end{array}\right).$$

The term $J_{weight}^{T}$ from Equation (35) adds as many columns as compliant actuators *j* are added to the end-effector.

• Open gripper:

A direct relationship between the energy that the actuator has to exert and the gripper position can be obtained by considering the total energy of cable 1:

(36) $$U_{1}=U_{1A}+U_{1B}=U_{1A}+U_{\min}+\left(∥F_{jBW}^{∥}∥+F_{jBA}\right)\delta_{1}.$$

With $U_{1A}$ chosen from the available values defined by (23) and $U_{\min},$ the initial energy stored in the mechanism is determined considering the potential energy reference when the spring from $j_{A}$ has its minimum energy. By combining (36) and (2), it is possible to obtain the position of the gripper with respect to the energy that the actuator of cable 1, *U_1_*, has to exert

(37) $$\delta_{1}=\delta_{1}\left(U_{1}\right)=\frac{-\left(∥F_{1BW}^{∥}∥+F_{jBA}\right)+\sqrt{{\left(∥F_{1BW}^{∥}∥+F_{jBA}\right)}^{2}+2K_{A}(U_{1}+U_{min})}}{K_{A}}$$

In this way, it is possible to know the energy needed by the actuator of cable 1 to set the desired position of gripper 1. However, previous restrictions already set in cable 1*_A_* have to be preserved. Those energy boundaries are defined as

(38) $$U_{1min}=U_{1Amin}+∥F_{1BW}^{∥}+F_{jBA}∥\delta_{1}$$

(39) $$U_{1max}=U_{1Amax}+∥F_{1BW}^{∥}+F_{jBA}∥\delta_{1}.$$

• Closed gripper:

When the gripper is closed or it is grabbing an object, the increasing energy of the gripper does not impose a movement but a force when the handled object is considered rigid. Due to the spring $K_{1B}$, the energy of this grabbing force can be directly added to the energy from the gripper weight by adding this term to (36). This expression acts when the grippers are closed ($\delta_{1}>L_{21}$):

(40) $$U_{1}=\left(U_{1A}+U_{\min}\right)+\frac{K_{B}{\left(\delta_{1}-L_{21}\right)}^{2}}{2}+\left(∥F_{jBW}^{∥}∥+F_{jBA}\right)L_{21}$$

where $L_{21}$ is the free range of displacement of the gripper before closing. The value of this length can be obtained by different methods like looking for the energy peaks and trend variations and vision or contact sensors. The structure matrices from Equation (32) do not vary because the tension increase in cable $1_{B}$ is compensated by the reaction force from the gripper to the main body. In this way, once the contact of the gripper with a rigid object is detected, the second part of Equation (40) acts and the force is controlled in the gripper instead of its position. The expression for the cable elongation is obtained in the same way as Equation (37)

(41) $$\delta_{1}=\frac{\left(K_{B}L_{21}+\sqrt{{\left(K_{B}L_{21}\right)}^{2}-2\Delta (K_{A}+K_{B})}\right)}{K_{A}+K_{B}}$$

with

(42) $$\Delta =\left(∥F_{1BW}^{∥}∥+F_{jBA}\right)L_{21}+\frac{K_{B}L_{21}^{2}}{2}-U_{1}+U_{min}.$$

• Gripper force analysis:

For the analysis of the closed state of the gripper, force values are more interesting than cable elongation. The force exerted by the gripper is defined as

(43) $$\begin{array}{c} F_{1BA}=K_{B}\left(\delta_{1}-L_{21}\right),\;\;\;\;if\;\delta_{1}>L_{21}\\ F_{1BA}\;=0,\;\;\;\;\;\;\;\;\;\;\;\;\;\;if\;\delta_{1}>L_{21} \end{array}$$

with the energy boundaries in function of force defined as

(44) $$U_{1min}=U_{Amin}+\frac{K_{A}L_{12}^{2}}{2}+\left(∥F_{jBW}^{∥}∥+F_{jBA}\right)L_{21}$$

(45) $$U_{1max}=U_{Amax}+\left(∥F_{jBW}^{∥}∥+F_{jBA}\right)L_{21}+\frac{F_{1BA}^{2}}{2K_{B}}$$

## 3. Theoretical Results

### 3.1. Energy Distribution of the Rigid End-Effector

The experiment consists of setting specific forces in the end-effector of a CDPR with eight cables, while two additional requirements are set for two different cables. 

The inertial frame of the robot is a parallelepiped of 10 × 5 m and a height of 6 m, and cables are deployed from the four upper corners of this frame. The end effector is a 150 kg cube with 1 m sides situated at the coordinates $\chi =\left(7,\;4,\;1.5\;|0,\;0,\frac{\pi }{6}\right)\left[m|\mathrm{rad}\right],$ seen from the fixed frame {F} and under the influence of the external force $F_{ext}=\left(200,\;0,\;-1470\;|-500,\;0,\;0\right)[N|\mathrm{Nm}]$ applied in the center of the cube.

**Figure 4:**
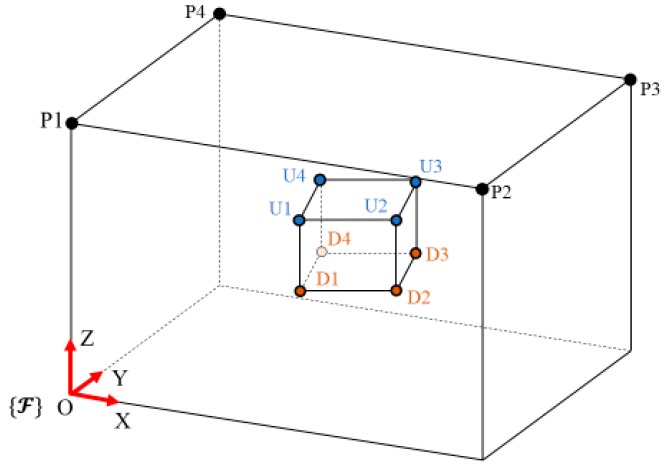
Cable-driven parallel robot scheme and the points for cable connections between the inertial frame and the end effector.

The connection of the cables from the inertial frame to the end effector have the following correspondence, according to Figure 4 and Table 1.

The results obtained for a robot with eight cables and six degrees of freedom are easy to show because they can be expressed in 3D space. 

**Figure 5:**
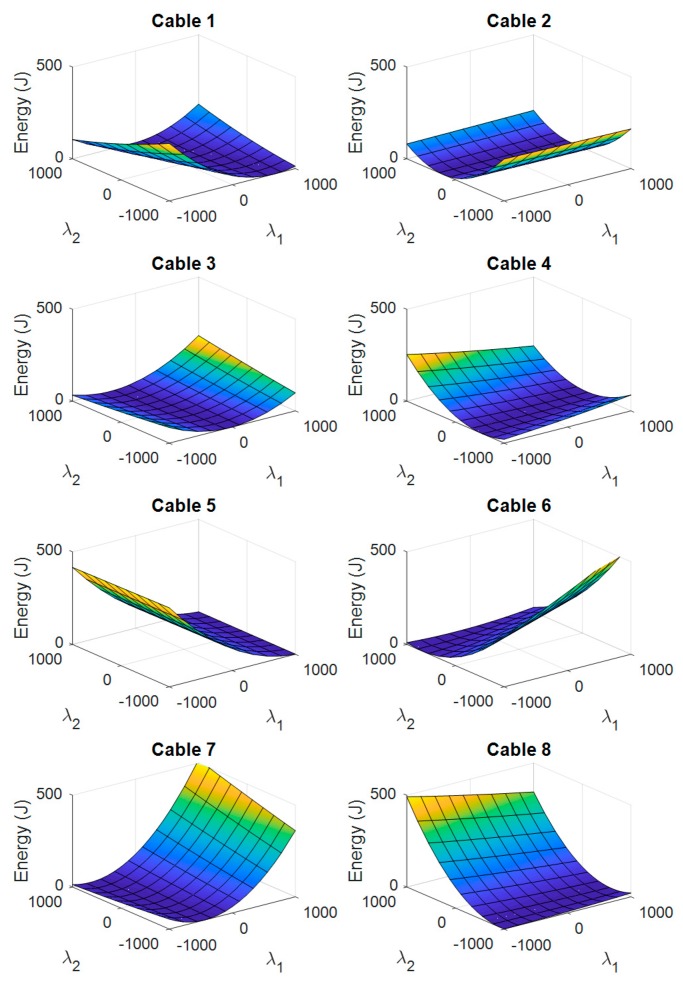
Surface corresponding to the potential energy of each cable as a function of the nullspace variables.

Depending on the nullspace position, the potential energy of each cable corresponds to a parabolic cylinder, as seen in Figure 5. The value of λ is the same for all cables, and the tension boundaries are not set yet.

**Figure 6:**
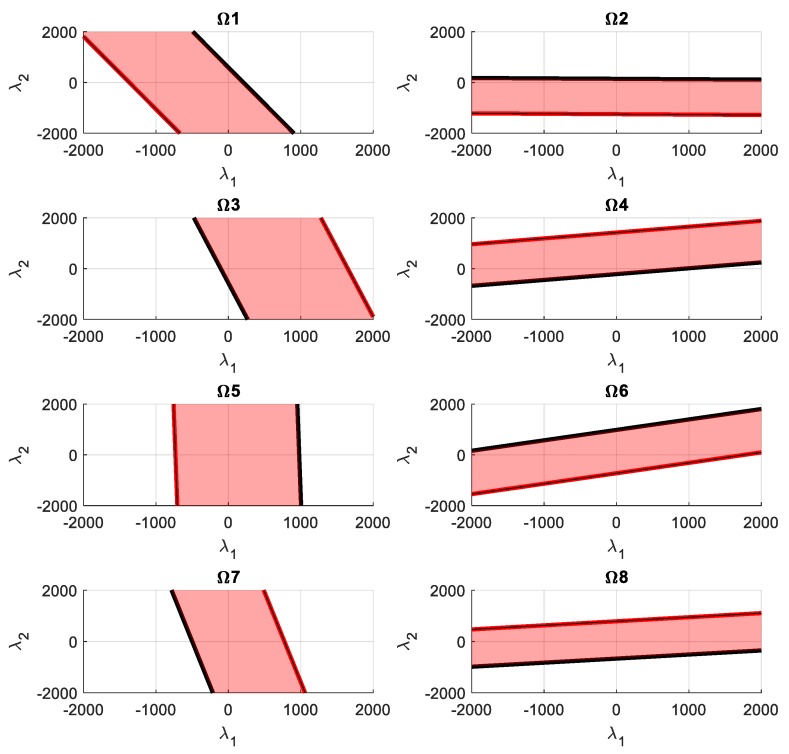
Red region $\left(\Omega_{i}\right)$: feasible region in the nullspace that provides a cable tension inside the allowable value. Black line $\left(\Lambda_{i}{}_{\min}\right)$: region where one or more cables achieve the minimum allowable tension value. Red line $\left(\Lambda_{i}{}_{\max}\right)$: region where one or more cables achieve the maximum allowable tension value.

The allowed tension values chosen for each cable are between 50 and 500 N. The spring stiffness is 10^3^ N/m. The boundary values for the energy are obtained with Equations (17) and (18), and they are 1.25 and 320 J. The regions of feasible energy for each cable $\left(\Omega_{i}\right)$ are obtained by using Equations (19) and (20). Those regions and their boundaries $\Lambda_{i}{}_{min}$ and $\Lambda_{i}{}_{max}$ are shown in Figure 6.

**Figure 7:**
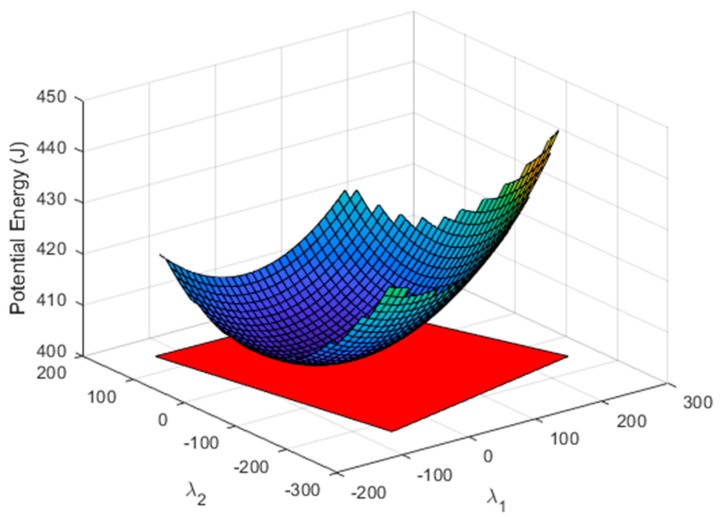
Surface section that represents the potential energy of all cables. The red polygon defines the region of the nullspace that provides feasible tension values in all cables.

The intersection of all the nullspace regions that define feasible tension values in each cable $\left(\Omega_{i}\right)$ provides the nullspace region (Ω), where all cables fulfil their tension conditions. The potential energy in all the cables was also obtained in (14). The representation of the total potential energy with the region of total feasible tension is shown in Figure 7. It is observed that its shape corresponds to an elliptic paraboloid. 

**Figure 8:**
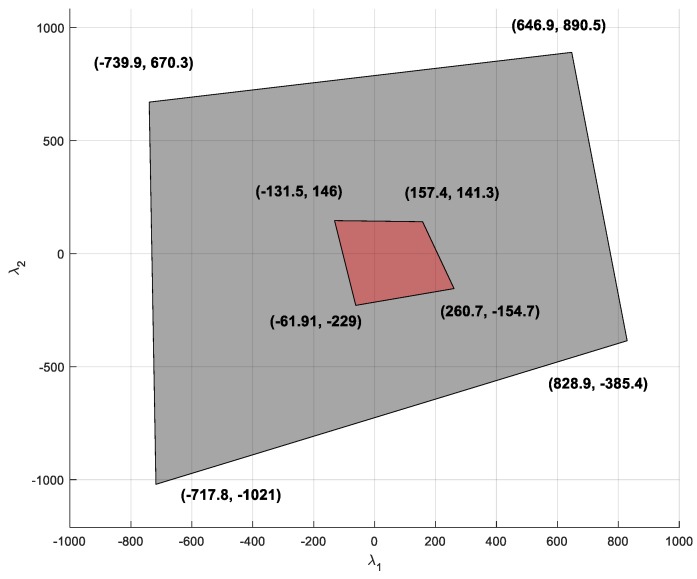
Red region (Ω): region of the nullspace that provides a feasible tension in all cables. Black region $\left(\Omega^{*}\right)$: region of the nullspace that provides feasible efforts in all the rigid links of a parallel robot with the same configuration of the cable robot.

It is also interesting to analyze those regions of maximum potential energy, $\left(\Lambda_{i_{\max}}\right)$, achieved with a negative tension in any link. A cable-driven robot cannot achieve this configuration, but it can be obtained by using a parallel robot with rigid links, which is able to exert compression efforts on the end effector. Figure 8 represents the feasible nullspace region for a cable-driven robot and the region for a parallel robot with rigid links under the same working conditions. The region corresponding to the cable is a subset of the region corresponding to the robot with rigid links. Only those regions where the boundary of the cable robot coincides with the boundary of to the rigid robot correspond to points where a link exerts maximum effort. So, in Figure 8, it is impossible to achieve the maximum tension in any cable before having another cable lose its tension under the lower limit.

In order to set the desired tension and deformation values in each cable, Equations (2) and (4) can be used. For this example, the desired linear deformation in cable 8 is 0.447 m, so following Equation (2), the energy of cable 8 is set to 100 J. 

**Figure 9:**
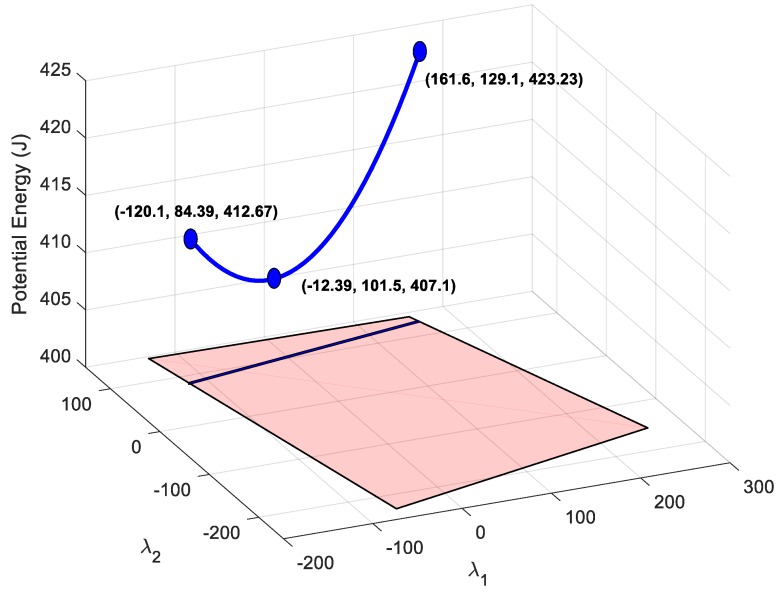
The blue 3D curve represents the potential energy stored in all cables that can be achieved by maintaining the condition of setting a deformation in the cable 8 of 0.2 m. The projection of this curve over the horizontal plane provides feasible nullspace variables. Coordinates are [$\lambda_{1}$, $\lambda_{2}$, *U*].

By imposing the energetic restriction in cable 8, its nullspace region of feasible tension collapses from a 2D surface to a 2D line. Figure 9 shows this line of feasible tension for achieving every restriction and the total elastic potential energy stored in the cables that depends on the nullspace variables.

**Figure 10:**
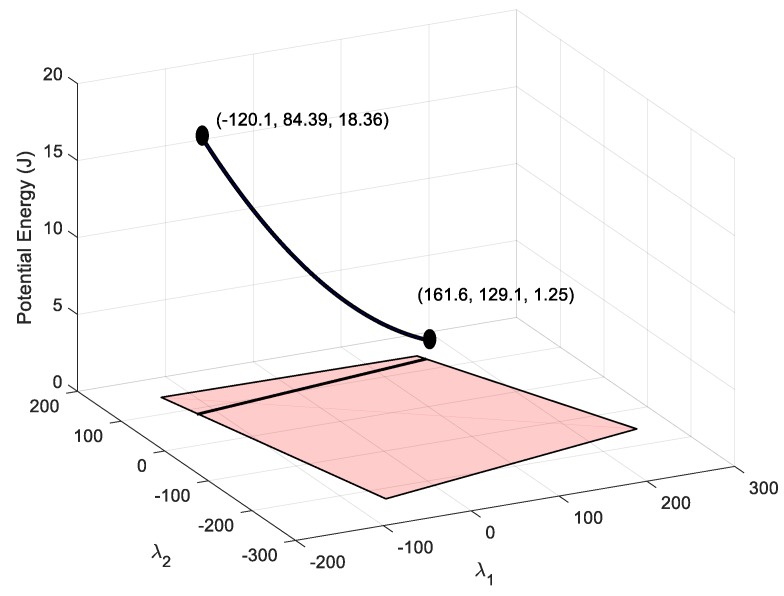
The black 3D curve represents the potential energy stored in cable 1 that can be achieved by maintaining the condition of setting a deformation in cable 8 of 0.2 m. The projection of this curve over the horizontal plane provides feasible nullspace variables. Coordinates are [$\lambda_{1}$, $\lambda_{2}$, U_1_].

With the remaining degree of freedom, it is possible to set the energy in another cable. For example, the range of potential energy available for cable 1 is obtained by substituting the values of the available nullspace variables in (9), which gives a range of $U_{1}\in \left(1.25,\;18.36\right)\;J$, as seen in Figure 10. This range of energy can be used to impose values of deformation in cable 1 according to (2): $\delta_{1}\in \left(50,\;191.6\right)10^{-3}\;m.$ Alternatively, it can be used to impose a tension value according to (4): $\tau_{1}\in \left(50,\;191.6\right)\;N$. If no restriction needs to be added to cable 1, the nullspace position for this cable could be that which minimizes the total potential energy. As seen in Figure 9, this position is $\lambda_{1}\in \left(-12.39,\;101.5\right),$ which provides a total potential energy of U=407.1 J.

In order to check the validity of the points to exert the desired force in the end-effector, three points of the valid line shown in Figure 9 are analyzed. By substituting the two extreme points and the minimum energy point of Figure 9 in Equation (1), the tension distribution is obtained. The tension distributions for each nullspace position are shown in Table 2.

As can be observed in Table 2, the tension in cable 8 is maintained at a constant level in order to provide the desired deformation of 0.447 m. It can be checked by substituting the tension value in energy Equations (4) and (2). In bold are the maximum and minimum tension values. Table 3 shows that the end-effector wrench is maintained. 

It can be seen also that the boundary points of the feasible line of the nullspace provide a tension value of 50 N in cable 3 (for the minimum $\lambda_{1}$ value) and in cable 1 (for the maximum $\lambda_{1}$ value). This is the minimum tension imposed for the cables.

The feasible nullspace is constrained by the linear elongation of cable 8, and the available tension in cable 1 is obtained. If higher range of tension in cable 1 is desired, one solution could be to set a less restrictive minimum tension in cable 1 or cable 3. 

### 3.2. Energy Distribution of the Reconfigurable End-Effector

**Figure 11:**
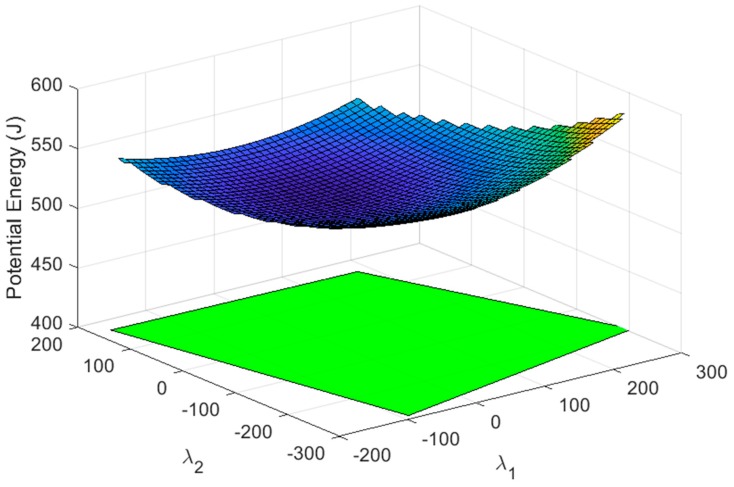
Surface section that represents the potential energy of all the cables when the two grippers are attached in cables 1 and 8. The green polygon defines the region of the nullspace that provides feasible tension values in all cables. It is different form the red polygon from Figure 7 because in this case the weight of the two grippers is being considered.

**Figure 12:**
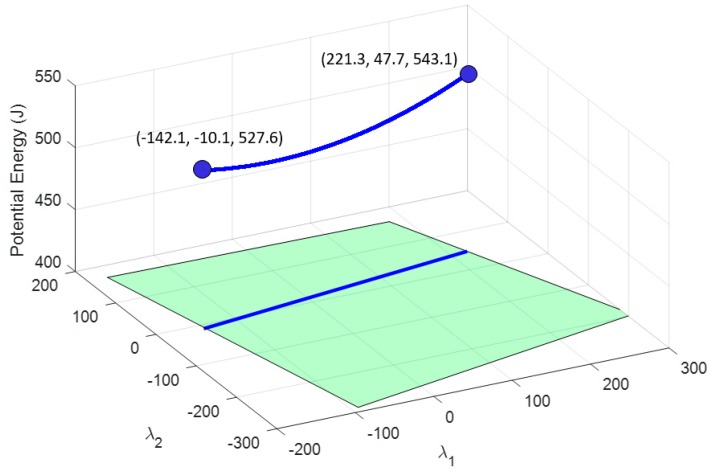
The blue 3D curve represents the potential energy stored in all cables that can be achieved by maintaining the condition of setting a deformation in cable 8 of 0.447 m and two grippers hanging from cables 1 and 8.

**Figure 13:**
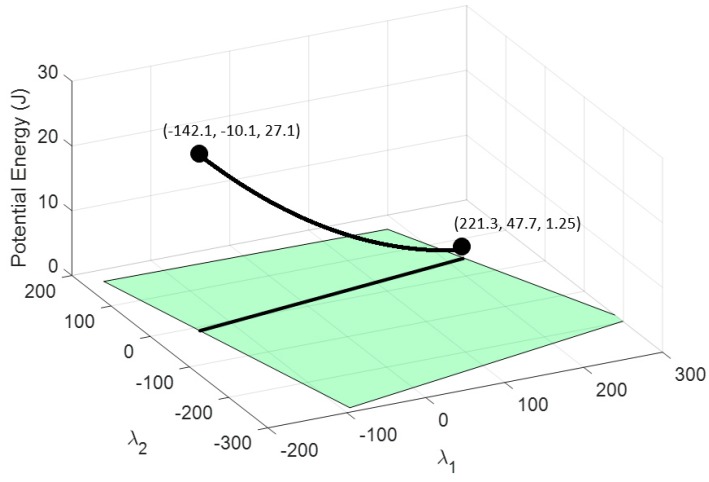
The black 3D curve represents the potential energy stored in cable 1_A_ by maintaining the condition of setting an elongation in cable 8 of 0.2 m and two grippers hanging from cables 1 and 8.

The example of the rigid end-effector is used to analyze the available energy in cable 1 when the elongation of cable 8 is set to a desired value. In this practical case, cables 1 and 8 have one compliant actuator each, as described in Figure 1. The structure matrix corresponding to the two grippers is

$$J_{weight_{1}}^{T}=\left(\begin{array}{c} \begin{array}{c} 0\\ 0\\ 1 \end{array}\\ \begin{array}{c} -0.183\\ -0.683\\ 0 \end{array} \end{array}\right)$$

$$J_{weight_{8}}^{T}=\left(\begin{array}{c} \begin{array}{c} 0\\ 0\\ 1 \end{array}\\ \begin{array}{c} 0.683\\ -0.183\\ 0 \end{array} \end{array}\right)$$

with $p_{1}=Rp_{1}^{M}={\left[-0.683,\;0.183,\;0.5\right]}^{T}\;m$ and $p_{8}=Rp_{8}^{M}=\left[-0.183,\;-0.683,\;-0.5\right]\;m$, $L_{11}=RL_{11}^{M}={\left[0,\;0,\;1.16\right]}^{T}\;m$ and $L_{18}=RL_{18}^{M}={\left[0,\;0,\;1.5\right]}^{T}\;m$. Considering $\;F_{1BW}=F_{8BW}=100\;N$, the external wrench imposed to the end-effector is

(46) $$F_{ext}^{*}=F_{ext}+J_{weight}^{T}\left(\begin{array}{c} \begin{array}{c} \begin{array}{c} F_{jBW}\\ \vdots \\ F_{rBW} \end{array} \end{array} \end{array}\right)$$

which, in this case, is $F_{ext}^{*}=[200,\;0,\;-1670\;|-550,\;-86.6,\;0]\;\left[N|\mathrm{Nm}\right]$. With this new external wrench applied to the end-effector, the proposed method is applied by upgrading the results from the previous section due to the consideration of the two grippers. The displacement of the cable $8_{A}$ is set and fixed to 0.447 m, which corresponds to the gripper being almost closed, because the length of the side of the end-effector is 1 m. The total potential energy considering the gripper is shown in Figure 11 while Figure 12 shows the total potential energy after imposing the displacement in $8_{A}$. The available energy in cable $1_{A}$ is $U_{1A}\in \left(1.25,\;27.1\right)\;J$, as seen in Figure 13, a higher range than in the case without grippers.

While the wrench applied to the end-effector by the cables is constant and the position of the gripper of cable 8 is fixed, the gripper of cable 1 can move in the range $\delta_{1}\in \left(50,\;232.8\right)10^{-3}\;m$. The aperture of this second gripper can be set by imposing the corresponding values in the nullspace.

• Open gripper

**Figure 14:**
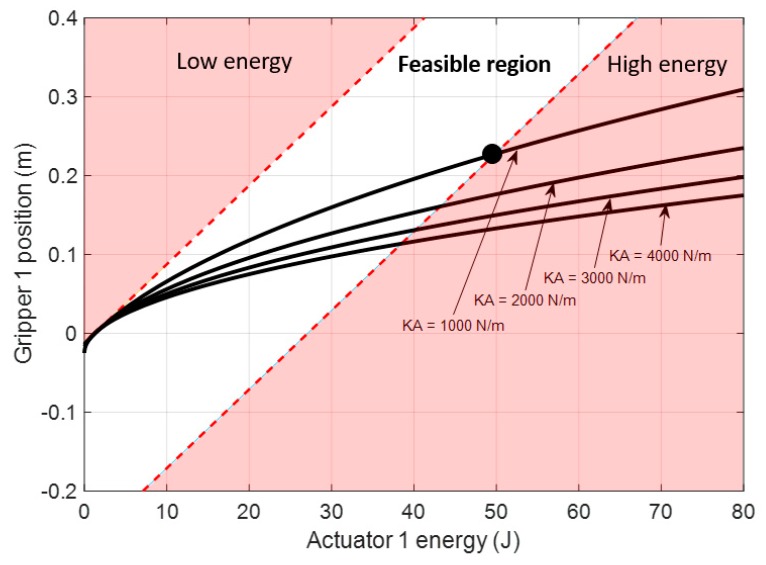
Feasible region of the energy imposed by actuator 1 for setting the position of the gripper attached to its compliant actuator. The range of feasible energy imposed by the actuator is related to the tension range available in the springs. Four different spring stiffness were analyzed for cable section $1_{A}$. Boundaries were applied for the case of $K_{A}=1000\;N/m$.

In Figure 14, the position of the gripper of cable 1 is set by imposing different values of available mechanical energy from actuator 1. As seen, the gripper initial position is obtained when the actuator provides 1.25 J in order to impose the minimum tension in the spring $K_{A}$ that makes it work inside its linear region. In this section the effect of the closed gripper is not analyzed. Different responses are obtained by considering different values of spring stiffness. Those results are obtained by the application of Equation (37) with the boundaries defined by Equations (38) and (39).

As observed in Figure 14, for a spring of $K_{A}=1000\;N/m$, the gripper range of motion is from 50 to 232.8 mm. 

• Closed gripper

**Figure 15:**
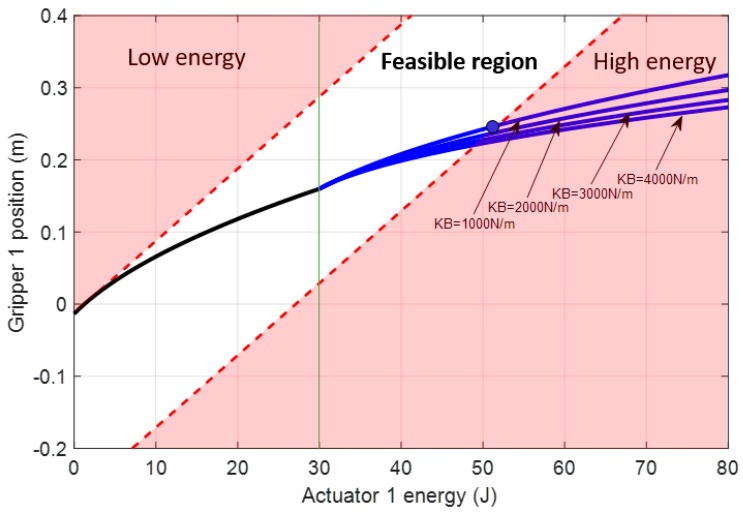
Feasible region of the energy imposed by actuator 1 for setting the position of the gripper attached to its compliant actuator without closing the gripper (black line) and with the gripper closed around a rigid solid (blue line). Four different springs were analyzed for cable section $1_{B}$. The contact was produced at 30 J, or $L_{21}=0.16\;m$ (green line), and consequently, the gripper was determined to be closed. Boundaries were applied for the case of $K_{B}=1000\;N/m$.

In this case, the effect of the closing gripper, which, in this case, appears when the gripper closes 0.16m from the initial state $\left(L_{21}=0.16\;m\right),\;\mathrm{is}\;\mathrm{analyzed}$. Those results, shown in Figure 15, are obtained by using Equations (41) and (42) with the same boundaries as the previous example defined by Equations (38) and (39).

• Gripper force analysis

**Figure 16:**
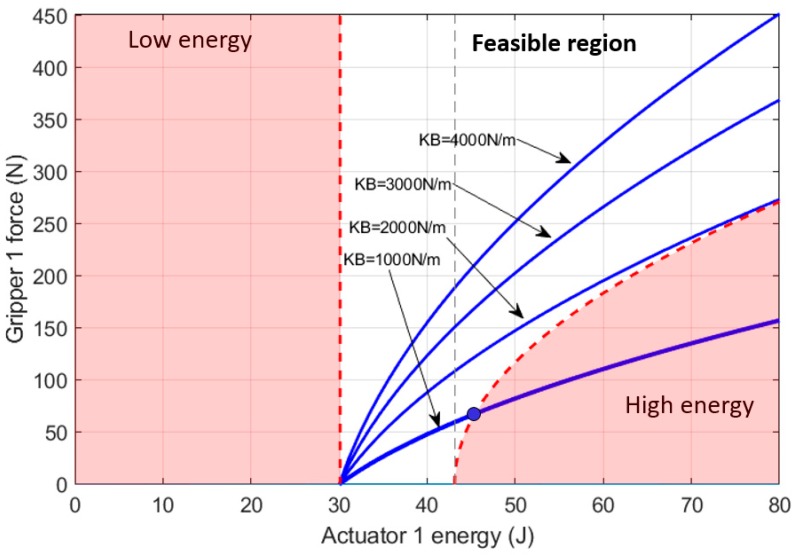
Feasible region of the energy imposed by actuator 1 for exerting force on the gripper attached to its compliant actuator. Four different springs were analyzed for cable section $1_{B}$. The boundaries were applied for the case of $K_{B}=1000\;N/m$, where the range of feasible force is $F_{1BA}\in \left(0,\;72.8\right)\;N$.

Once the gripper is closed, the energy method allows the force that the gripper is exerting to be measured. The results shown in Figure 16 show the relationship between this force and the energy of the actuator. Those results were obtained from Equation (43) and the force boundaries were defined by Equations (44) and (45).

## 4. Simulated Results

### 4.1. Rigid Solid End-Effector

**Figure 17:**
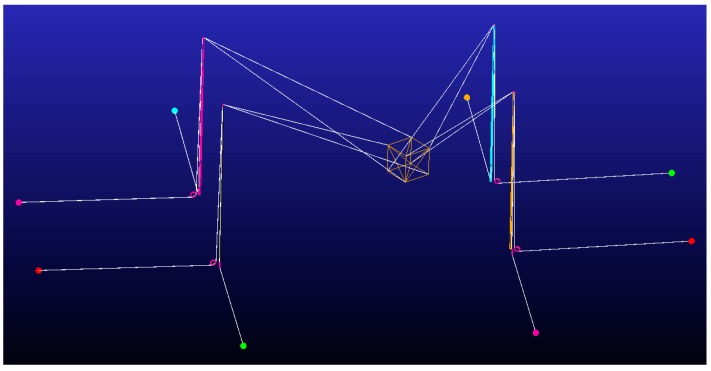
Dynamic multibody model tested in MSC ADAMS. Table 4 defines the robot dimensions and configuration. Cables are actuated by pulling from their ends situated in the basement. Those cables have to pass around two pulleys, one situated in the pillar base and the other in the top of the pillar.

**Figure 18:**
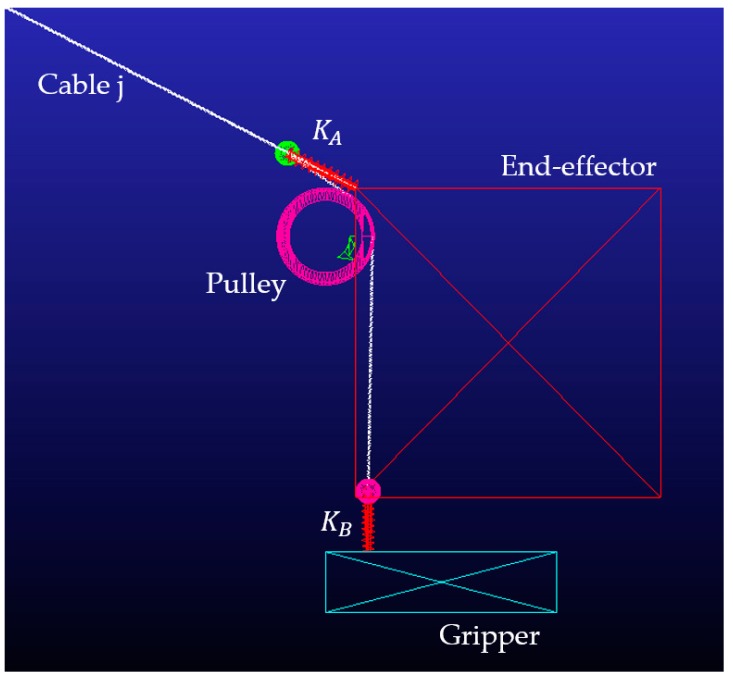
Dynamic model of one of the grippers. By pulling “Cable j”, the spring $K_{A}$ elongates and the gripper begins to close, moving towards the end-effector. This gripper moves along a linear guide, and the secondary cable moves inside the end-effector. The pulley is designed to align this cable in a vertical way.

In order to check the validity of the method and the theoretical results, a CDPR was modeled by using the multibody dynamic software MSC ADAMS. The robot has been modeled with the same dimensions, positions, and requirements imposed in the previous section. In this model the cable mass is included, and friction effects, pulley dynamics, and a linear spring are attached between the cables and the end-effector. Figure 17 and Figure 18, and Table 4 show, in detail, the structure, size, and forces applied. 

A tension planner was defined with the end-effector fixed in its position. By fixing the end-effector, this analysis focused only on forces acting on the robot. In real experiments, position control must be performed to maintain the desired position. 

The tension planner goes from the nullspace point where cable 1 has minimal energy until the nullspace point that provides maximal energy to cable 1. This trajectory can be observed in Figure 13.

**Figure 19:**
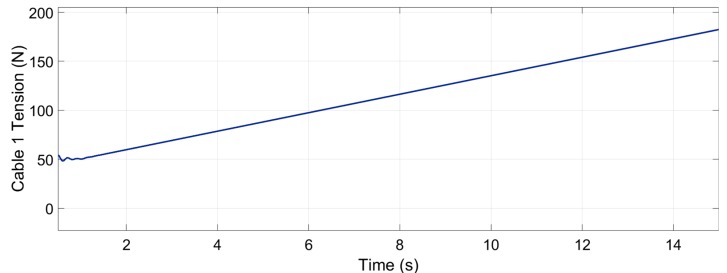
Tension in cable 1—from 50 N to 182.4 N.

Figure 19 shows how the tension in cable 1 increased from the minimum tension, that is 50 N, until 182.4 N. The maximum desired tension was 191.6 N. This error in the maximum tension of 9.4 N could have come from the weight and inertia of the cables, which were not taken into account.

**Figure 20:**
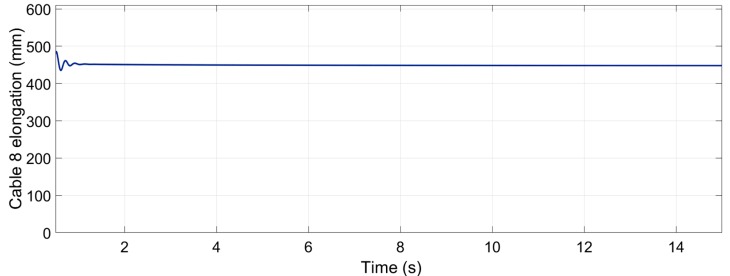
Elongation of the spring situated between cable 8 and the end-effector. The value oscillates between 0.451 and 0.448 m after the stabilization of the measure.

Figure 20 shows that the elongation of cable 8 m varied from 0.448 to 0.451 m. The desired elongation was 0.447, so the dynamic experiment showed a small reduction in this elongation that could be produced by the cable weight and inertia.

**Figure 21:**
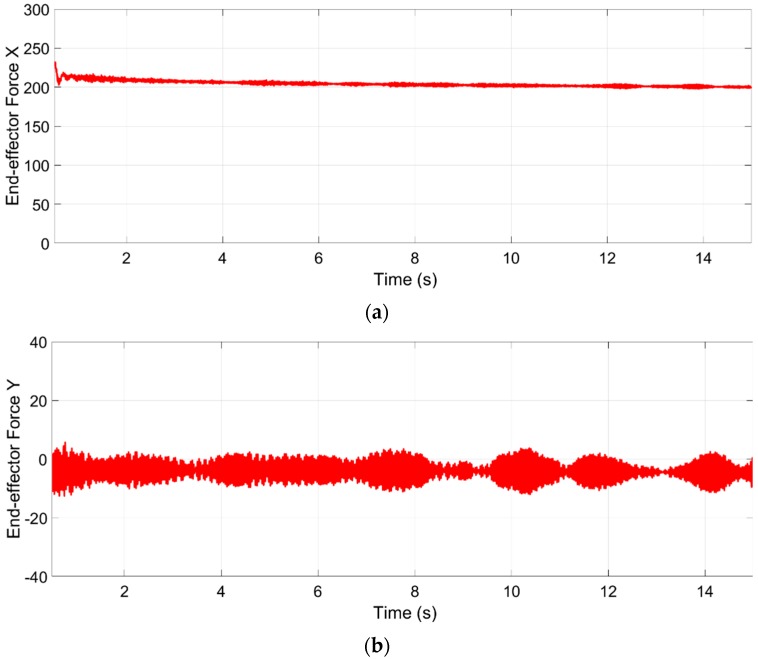

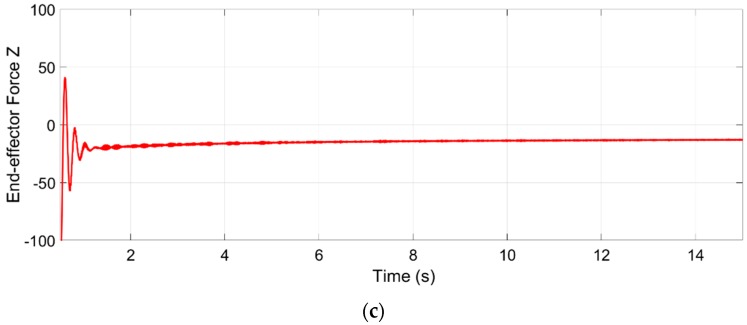
Forces acting on the end-effector. (**a**) Force in the *x*-axis from 215 to 199 N. (**b**) Force in the *y*-axis from 5.75 to −12 N. (**c**) Force in the Z axis from −22.8 to −13.3 N.

The resultant forces in the end-effector, shown in Figure 21, demonstrate that the end-effector is maintained under the desired forces, even when the additional two conditions have been set in cables 1 and 8. The measured force in the Z axis should be zero, because the desired force in this axis is equal to the end-effector weight. 

### 4.2. Reconfigurable End-Effector

In the previous subsection, the results from the energy distribution along the eight cables of the CDPR were obtained to set the desired values of tension and deformation in the redundant cables. In this subsection, those results are used to set the desired values of the gripper to determine its aperture range and the current position that corresponds to the energy consumed by the corresponding actuator. 

**Figure 22:**
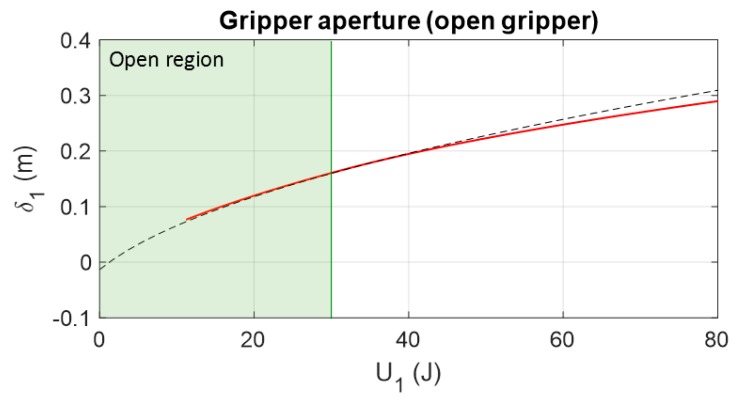
Comparison between the theoretical and simulated gripper aperture (measuring the value of $L_{21}$) and the energy of actuator 1. At 30 J or 0.16 m, the gripper closes, increasing the error between the theoretical and simulated results.

Figure 22 shows the aperture of the gripper (value $L_{21}$) considering Equation (37) along all values of actuator energy. The green region is the region where the gripper is not closed yet. It is observed that the error between the experimental and theoretical results increases after closing the gripper. 

**Figure 23:**
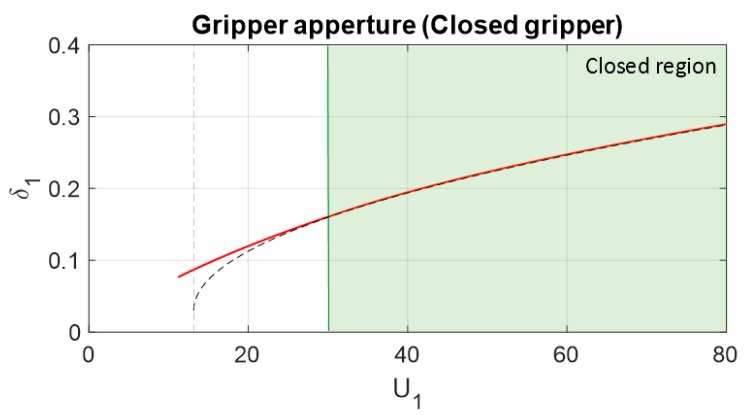
Comparison between the theoretical and simulated actuator displacement compared with the actuator energy when the gripper is already closed.

Figure 23 shows the displacement of the cable in the actuator in comparison with the energy of the actuator when the gripper is already closed. This displacement increases the potential energy of both springs of the compliant mechanism of the cable. Knowing this displacement, it is possible to determine the energy or force applied to the object grasped. 

**Figure 24:**
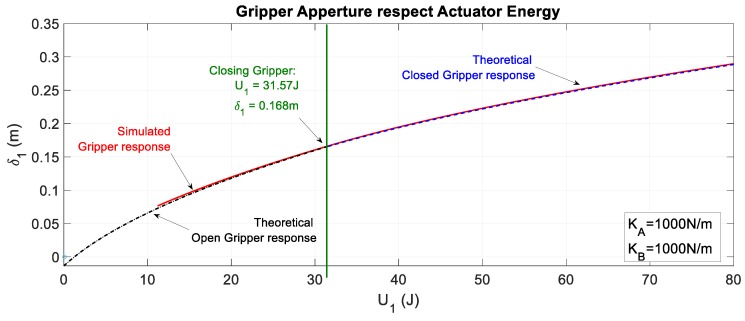
Comparison between the theoretical and simulated displacement of the cable in the actuator when the gripper is open or closed by considering the two different expressions.

Figure 24 considers both behaviors: the opened and closed gripper.

## 5. Discussion

Taking advantage of the actuation redundancy of parallel robots allows the workspace and the performance to increase, and in this case, it allowed secondary tasks to be performed if desired. The new perspective proposed in this article is a potential method of energy analysis that considers spring deformation, cable tension, and weights. A close-form equation was developed to relate the potential energy of each cable or the total potential energy with the nullspace that characterizes the redundancy effect. 

The two appendices describe the analysis of the closed-form equation of the energy in order to demonstrate that the potential energy values along the nullspace maintain the same topology for any wrench or position value. 

The theoretical and simulated results of the selection of desired tension and deformation were compared in two different cables maintaining the same wrench on the end-effector. The simulated results show that the tension applied to the end-effector and the deformation of the springs was slightly lower than the theoretical value. This error could be due to the weight and inertia of the cable, which are not considered in the energetic model. Those parameters take a portion of the energy provided by the actuators. Future work could analyze this effect in order to develop a more detailed expression of energy. 

Wrench variations in the end-effector when the nullspace values vary are low enough to be handled by one of the well-known position controls of parallel robots. In this article, an analysis of the energy distribution along the cables is performed by fixing the position of the end-effector. In this way, wrench errors are analyzed without the perturbation of variations of the Jacobian matrix.

The second part of the article describes a mechanism based on previous results. This mechanism substitutes the rigid body of the previous end-effector to perform grasping tasks with r grippers. Gripper movement is related to the spring deformation of the rigid end-effector, and gripper force is related to cable tension.

Some upgrades of the usual Jacobian matrix have been developed to include the effects of the additional grippers’ weights, whose effect on the main body of the end-effector varies with the orientation of the mechanism.

The comparison between the theoretical and simulated results shows that the method for measuring the gripper’s position by using energy is mainly suitable for those applications where the end-effector cannot have any kind of sensor or actuator that is able to provide us with a valid measurement. Some of those scenarios could be underwater environments, extreme temperatures, and emplacements like fires or outer space.

In future research, it could be interesting to consider the relation between the energy of the actuators and the position/force of the grippers in order to make an energy-based control as seen in [38]. This approach could also allow us to obtain a better vision and to help us to design and control hyper-redundant elastic parallel robots by imposing as many additional conditions in cables as degrees of redundancy that the system has. Also, it would be interesting to conduct visual feedback from outside the workspace to close the control loop of the gripper [39], reaching more complex grasping processes. 

## 6. Conclusions

In this study, a method for selecting the potential energy distribution in a redundant cable-driven parallel robot was developed. With this method, it was possible to set the desired values of linear deformation or tension in redundant cables as well as to check the range of available energy in each cable to set these conditions. 

Energy from redundant cables was used in secondary tasks consisting of moving two grippers attached to two cables. The range of motion and the range of force of these grippers was obtained based on the available energy. This energy consideration allowed the cable elongation, tension, and weight effects to be analyzed. 

It was also possible to analyze the shapes of the energy curves and to define the directions of those boundaries that can be related to energy values like tension or deformation. This method can be used for any number of cables and degrees of redundancy.

## 7. Patents

“Robot Paralelo actuado mediante cables tirantes con efector reconfigurable” ES2687712(A1)–2018-10-26.

## Figures and Tables

**Table 1:** Connection points for cables R = 2.

Inertial Frame	End-Effector	Cable Name
P1	U4, D2	Cable1, Cable2
P2	U1, D3	Cable3, Cable4
P3	U2, D4	Cable5, Cable6
P4	U3, D1	Cable7, Cable8

**Table 2:** Tension distribution for each nullspace position ($\delta_{8}=0.447\;m)$.

Nullspace Position	Tension Distribution (N)
(−120.1, 84.39)	[191.6, 83.1, **50.0**, 199.7, 531.4, 422.3, 280.3, **447.2**]
(−12.39, 101.5)	[137.5, 72.9, 97.4, 196.2, 484.0, 434.23, 345.23, **447.2**]
(161.6, 129.1)	[**50.0**, 56.5, 174.0, 190.5, 407.5, 453.5, 450.0, **447.2**]

**Table 3:** End-effector wrench for each tension distribution ($\delta_{8}=0.447\;m)$.

Nullspace Position	Total Potential Energy (J)	Cable 1 Potential Energy (J)	End-Effector Wrench (N|Nm)
(−120.1, 84.39)	412.67	18.36	[200, 0, −1470|−500, 0, 0]
(−12.39, 101.5)	407.1	9.45	[200, 0, −1470|−500, 0, 0]
(161.6, 129.1)	423.23	1.25	[200, 0, −1470|−500, 0, 0]

**Table 4:** Cable-driven robot parameters.

Parameter	Value
Frame dimensions (X, Y, Z) [m]	[10, 5, 6]
End-effector mass [kg]	150
Gripper mass [kg]	10.2
End-effector center position (X, Y, Z, R, P, Y) [m, rad]	[7, 4, 1.5, 0, 0, $\frac{\pi }{6}$]
External wrench (F_X_, F_Y_, F_Z_, M_X_, M_Y_, M_Z_) [N, Nm]	[200, 0, −1470, −500, 0, 0]

## Appendix A

Demonstration that the topology of the *i*-cable energy corresponds to a parabolic cylinder when any two different nullspace variables are considered.

By expressing (9) with homogeneous coordinates, the following simplified expression is obtained:

(A1) $${\left(\lambda_{h}\right)}^{T}M_{i}\lambda_{h}=0$$

with

(A2) $$\lambda_{h}=\left(\begin{array}{c} 1\\ \lambda_{\alpha }\\ \lambda_{\beta }\\ U_{i} \end{array}\right)$$

(A3) $$M_{i}=\frac{1}{2k_{i}}\left(\begin{array}{cccc} b_{i}^{2} & b_{i}a_{i\alpha } & b_{i}a_{i\beta } & -k_{i}\\ b_{i}a_{i\alpha } & a_{i\alpha }^{2} & a_{i\alpha }a_{i\beta } & 0\\ b_{i}a_{i\beta } & a_{i\alpha }a_{i\beta } & a_{i\beta }^{2} & 0\\ -k_{i} & 0 & 0 & 0 \end{array}\right)$$

The classification of quadrics is done according their signature, σ, defined as the module of the difference between the number of positive and negative eigenvalues of the adjunct matrix of $M_{i_{00}}$, with $M_{i_{00}}$ being the first element of $M_{i}$:

(A4) $$M_{i_{00}}=\frac{1}{2k_{i}}\left(\begin{array}{ccc} a_{i\alpha }^{2} & a_{i\alpha }a_{i\beta } & 0\\ a_{i\alpha }a_{i\beta } & a_{i\beta }^{2} & 0\\ 0 & 0 & 0 \end{array}\right).$$

The eigenvalues, *u*, of $M_{00}$ can be obtained by using the associated invariants:

(A5) $$\det\left(uI_{3}\right)=u^{3}-Iu^{2}+IIu^{3}-III$$

with

(A6) $$I=a_{i\alpha }^{2}+a_{i\beta }^{2}+0\geq 0$$

(A7) $$\mathrm{II}=det\left(\begin{array}{cc} a_{i\alpha }^{2} & a_{i\alpha }a_{i\beta }\\ a_{i\alpha }a_{i\beta } & a_{i\beta }^{2} \end{array}\right)+det\left(\begin{array}{cc} a_{i\alpha }^{2} & 0\\ 0 & 0 \end{array}\right)+det\left(\begin{array}{cc} a_{i\beta }^{2} & 0\\ 0 & 0 \end{array}\right)=0$$

(A8) $$\mathrm{III}=\det\left(M_{i_{00}}\right)=0$$

Considering (22), there are two eigenvalues that are equal to zero. The determinant of M can define the shape of the quadric:

(A9) $$\det\left(M_{i}\right)=k_{i}det\left(\begin{array}{ccc} b_{i}a_{i\alpha } & a_{i\alpha }^{2} & a_{i\alpha }a_{i\beta }\\ b_{i}a_{i\beta } & a_{i\alpha }a_{i\beta } & a_{i\beta }^{2}\\ -k_{i} & 0 & 0 \end{array}\right)=0$$

Because the determinant is null, new invariants need to be introduced:

(A10) $$II^{\prime }=\det\left(\begin{array}{cc} b_{i}^{2} & b_{i}a_{i\alpha }\\ b_{i}a_{i\alpha } & a_{i\alpha }^{2} \end{array}\right)=0$$

(A11) $$III^{\prime }=\det\left(M_{i_{11}}\right)+\det\left(M_{i_{22}}\right)+\det\left(M_{i_{33}}\right)=-k_{i}\left(a_{i\alpha }^{2}+a_{i\beta }^{2}\right)\leq 0$$

The value of *II* is zero and *III’* is less or equal to zero, so the shape of the quadric that corresponds to the potential energy stored in the cable in function of two nullspace variables is a parabolic cylinder.

## Appendix B

Demonstration that the topology of the total energy stored in all cables corresponds to an elliptic paraboloid when any two different nullspace variables are considered.

A similar method to that described in Appendix A is used. The equation with homogeneous coordinates is

(A12) $${\left(\lambda_{h}^{*}\right)}^{T}M\lambda_{h}^{*}=0$$

with

(A13) $$\lambda_{h}^{*}=\left(\begin{array}{c} 1\\ \lambda_{\alpha }\\ \lambda_{\beta }\\ U \end{array}\right)$$

(A14) $$M=\sum_{i=1}^{m}M_{i}.$$

The invariants are

(A15) $$I=\sum \frac{a_{i\alpha }^{2}}{2k_{i}}+\sum \frac{a_{i\beta }^{2}}{2k_{i}}+0>0$$

(A16) $$\mathrm{II}=det\left(\begin{array}{cc} \sum \frac{a_{i\alpha }^{2}}{2k_{i}} & \sum \frac{a_{i\alpha }a_{i\beta }}{2k_{i}}\\ \sum \frac{a_{i\alpha }a_{i\beta }}{2k_{i}} & \sum \frac{a_{i\beta }^{2}}{2k_{i}} \end{array}\right)=0$$

(A17) $$\mathrm{III}=\det\left(M_{00}\right)=0$$

The determinant of **M** is

(A18) $$\det\left(M\right)=det\left(\begin{array}{ccc} \sum \frac{b_{i}a_{i\alpha }}{2k_{i}} & \sum \frac{a_{i\alpha }^{2}}{2k_{i}} & \sum \frac{a_{i\alpha }a_{i\beta }}{2k_{i}}\\ \sum \frac{b_{i}a_{i\beta }}{2k_{i}} & \sum \frac{a_{i\alpha }a_{i\beta }}{2k_{i}} & \frac{\sum a_{i\beta }^{2}}{2k_{i}}\\ -\frac{1}{2} & 0 & 0 \end{array}\right)=\frac{1}{2}\left({\left(\sum \frac{a_{i\alpha }a_{i\beta }}{2k_{i}}\right)}^{2}-\sum \frac{a_{i\alpha }^{2}}{2k_{i}}\sum \frac{a_{i\beta }^{2}}{2k_{i}}\right)\neq 0$$

In order to classify the quadric, it is necessary to know the sign of the det(M). It only can be zero if $a_{i\alpha }=a_{i\beta }$. This condition is never achieved because the kernel of A cannot lose rank, considering the rank-nullity theorem [40]. Thus, because III = 0 and I > 0, the shape of the total energy space when varying two nullspace variables is a elliptic paraboloid.
